# Effect on the mechanical properties of type I collagen of intra-molecular lysine-arginine derived advanced glycation end-product cross-linking

**DOI:** 10.1016/j.jbiomech.2017.11.021

**Published:** 2018-01-23

**Authors:** T.A. Collier, A. Nash, H.L. Birch, N.H. de Leeuw

**Affiliations:** a Institute of Natural and Mathematical Sciences, Massey University, Auckland 0632, New Zealand; b Department of Physiology, Anatomy and Genetics, University of Oxford, South Parks Road, Oxford OX1 3QX, United Kingdom; c Institute of Orthopaedics and Musculoskeletal Science, UCL, RNOH Stanmore Campus, London, United Kingdom; d School of Chemistry, Cardiff University, Cardiff CF10 1DF, United Kingdom

**Keywords:** Collagen, Molecular dynamics, Ageing, Glycation, Protein cross-linking, Molecular biomechanics

## Abstract

Non-enzymatic advanced glycation end product (AGE) cross-linking of collagen molecules has been hypothesised to result in significant changes to the mechanical properties of the connective tissues within the body, potentially resulting in a number of age related diseases. We have investigated the effect of two of these cross-links, glucosepane and DOGDIC, on the tensile and lateral moduli of the collagen molecule through the use of a steered molecular dynamics approach, using previously identified preferential formation sites for intra-molecular cross-links. Our results show that the presence of intra-molecular AGE cross-links increases the tensile and lateral Young’s moduli in the low strain domain by between 3.0–8.5% and 2.9–60.3% respectively, with little effect exhibited at higher strains.

## 1 Introduction

Collagen is one of the major contributors to the mechanical properties of mammalian tissues. There are currently 28 different human collagen types (Kadler et al., 2007), with the fibril-forming type I collagen being the predominant form. Type I collagen is typically found in connective tissues such as tendon, ligament, bone, skin and the cornea of the eyes of vertebrates (Bhattacharjee and Bansal, 2005). Type I collagen provides not only the tensile strength to these connective tissues, but it also serves as a structural framework for the attachment of cell and other extra-cellular matrix (ECM) biomolecules (Sweeney et al., 2008). The functional integrity of collagen within the collagenous tissues is vital for the normal functioning of the body.

With collagen making up a significant proportion of connective tissues, approximately 90% in some cases (Kannus, 2000), its biomechanical and energy storage properties are of utmost importance (Franchi et al., 2007). The mechanical functions of the supramolecular structure in collagenous tissues are optimised for the direction and magnitude of load. Tendons have uni-directional tensile strength, a consequence of fibre alignment in thick bundles parallel to the long axis of the tendon (Silver et al., 2003). In skin, the fibres form an isotropic network capable of managing multi-directional forces (Ottani et al., 2001). The forces experienced by the collagenous tissues vary greatly in magnitude and direction. Applied forces can be sporadic, sustained or repetitive. For example, a runner’s Achilles tendon can experience peak forces of 11.4 times their body weight (Dixon and Kerwin, 2002), bearing over 2000 cyclic loading events during a 5 km run (Lichtwark et al., 2013).

The hierarchical structure of the collagen supra-molecular assembly results in several mechanical properties over different scales: the molecular scale, i.e. the response of the collagen molecule to strain; the fibrillar scale, with the response of fibrils to an applied load; the microscale, which incorporates the response of a collagen fibre; and finally the macroscale, where the mechanics of the whole collagenous tissue are considered. At the molecular scale, a number of atomistic and coarse-grained molecular dynamics simulations have been conducted to probe the response of the molecule to an applied load (Bhowmik et al., 2007, Buehler, 2006, Gautieri et al., 2009), with a small number of experimental studies probing single molecule responses to a load (Bozec and Horton, 2005, Sun et al., 2002). At the microfibril and fibril level the amount of experimentally determined data increases, with experiments using a wide variety of techniques such as a microelectromechanical systems device (Eppell et al., 2006), X-ray diffraction (Sasaki and Odajima, 1996a) and AFM (Van Der Rijt et al., 2006, Wenger et al., 2007). The results of these studies have shown that as the hierarchical scale increases, Young’s modulus (YM) decreases significantly, with the molecular level ranging from 2 to 9 GPa, whereas on the tissue scale, the modulus varies between 0.001 and 1 GPa, depending on the tissue type (Sherman et al., 2015). The most likely reason for this variation is the inter-fibrillar sliding and the straightening and reorientation of the fibrils/fibres (Parry, 1988).

Due to the size and complexity of collagen molecules, to date, very few computational investigations have been conducted. Most investigations have utilised collagen-like peptides (CLPs), which are triple helical peptides of typically 30 residues in length. CLPs have similar properties to collagen and due to their reduced size are more amenable to study. The first computational study to probe the mechanical properties of the collagen molecule was conducted by Lorenzo and Caffarena (2005), when they conducted a steered molecular dynamics approach, testing the molecular response of short 29–30 amino acid collagen-like peptides, based on a model with two springs in series (Lorenzo and Caffarena, 2005). However, this study used a polarisable continuum model, which was shown to be insufficient in a later study on the participation of structural water in carrying load (Zhang et al., 2007), thereby altering the mechanical properties. Two other studies have been conducted since, investigating several elements, e.g. how helical hierarchy controls collagen deformation (Pradhan et al., 2012) and the role of the mature enzymatic cross-links (Kwansa et al., 2014). Yet to date, no such study has been conducted to investigate the effect that age-related non-enzymatic cross-links have on the mechanical properties of collagen.

Enzymatic cross-links are initiated by the enzyme lysyl oxidase and form inter-molecularly between the collagen molecules at defined locations, to provide functional stability to the collagen fibril (Reiser et al., 1992). Unlike enzymatic cross-links, non-enzymatic cross-links are considered pathological, disrupting normal biological function and altering the mechanical properties of the tissue (Reddy, 2004, Schalkwijk et al., 2004). Non-enzymatic cross-links, most commonly advanced glycation end products (AGEs), are formed through a series of successive chemical reactions between a reducing sugar, such as glucose (an aldose) or fructose (a ketose), and a protein or lipid (Biemel et al., 2001, Vistoli et al., 2013). To date only a few physiologically relevant AGEs have been characterised from tissues ex vivo, most notably lysine-lysine and lysine-arginine cross-link forming AGEs (Biemel et al., 2002). The concentration of AGEs have been shown to steadily increase with normal ageing (Simm et al., 2007, Thorpe et al., 2010), particularly in tissues with low turnover rates, such as type I collagen whose half-life can be up to 200 years in tendon (Heinemeier et al., 2013). Studies have shown that the mechanical and biological functions of collagen are disrupted or altered upon formation of AGE cross-links, which may account for some of the age-related mechanical decline of collagenous tissues (Monnier et al., 2005). Reddy et al. found that *in vitro* incubation of rabbit Achilles tendon in ribose increased levels of the AGE pentosidine, as well as increasing the YM by 159%, from 24.89 ± 1.52 MPa to 65.087 ± 14.41 MPa, suggesting that the presence of AGE cross-links increases the stiffness of soft tissue (Reddy, 2004).

**Fig. 1:**
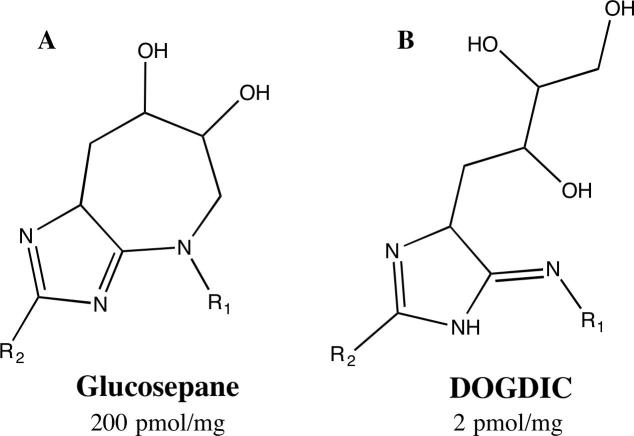
Schematic image of Lysine (R_1_) and Arginine (R_2_) Cross-linking AGEs, (A) Glucosepane, (B) DOGDIC, with their concentration in human albumin serum as determined by Biemel et al., reported below each structure (Biemel et al., 2002).

In previous work, we have identified the preferential intra-molecular DOGDIC and glucosepane AGE formation sites within the collagen molecule, and discussed their potential impact on the biological function of collagenous tissues (Collier et al., 2016, Collier et al., 2015). Glucosepane is the most abundant lysine-arginine derived cross-linking AGE, forming a 7 membered ring containing two hydroxyl groups (Fig. 1A). DOGDIC is a hexose-derived lysine-arginine cross-linking AGE, which forms a 5-membered ring at the guanidine functional group of arginine, with an aliphatic carbon chain extending out which contains three hydroxyl groups, as seen in Fig. 1B. In this work we aim to expand upon those findings by probing, through the use of steered molecular dynamics simulations, the effect of the presence of these cross-links at the identified sites on the mechanical properties of the collagen molecule. We expect that the presence of an AGE cross-link within the collagen molecule will result in an increase in stiffness, due to interference with the normal extension mechanism.

## 2 Results and discussion

Using a steered molecular dynamics (MD) approach we have probed the effect of AGE cross-linking on the tensile and lateral mechanical properties of the collagen molecules. Sets of wild type (native) and cross-linked collagen-like peptides (CLPs) were created by taking short collagen sections from our previous simulations, 7 residues either side of the eleven identified preferential intra-molecular cross-linking locations (Collier et al., 2016, Collier et al., 2015). Six constant velocity steered MD simulations (n = 6) were conducted to probe the mechanical properties along the principal axis of the collagen molecule and transverse to the principal axis.

The YM for the models were then calculated in two strain regions, low strain (0–15%) and intermediate strains (20–40%). Values are reported for the eleven different CLPs as relative differences between the wild type and cross-linked models. Relative values will remove the effect of variation in the primary sequence on the reported mechanical properties (Uzel and Buehler, 2009). The absolute values for the tensile YM of the wild-type collagen varied between 3 and 4.5 GPa, which is in good agreement with previously reported values (Sherman et al., 2015).

**Fig. 2:**
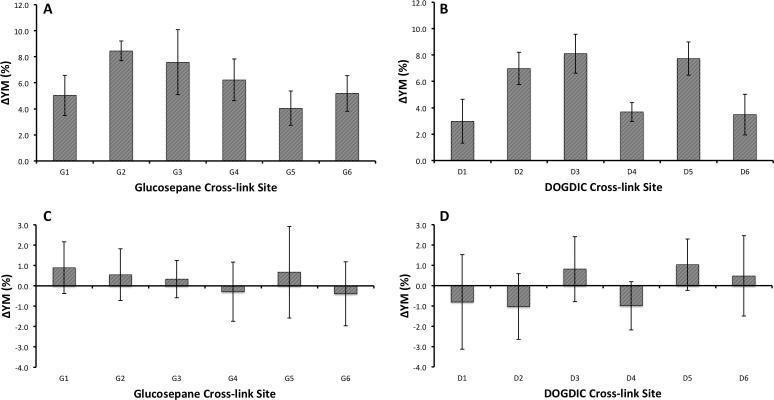
The percentage change in the tensile Young’s modulus upon the formation of a Glucosepane (A, C) or DOGDIC (B, D) cross-link relative to the wild type collagen, in the low strain (A, B) region (0–15% strain) and in the intermediate strain (C, D) region (20–40% strain). The error bars illustrate the uncertainty as calculated by the standard error of the mean in the calculated values, for the 6 repeats. G (glucosepane) and D (DOGDIC) numbering corresponds to the numbering for sites given in Table 1.

In the low strain domain an increase in the calculated value for the tensile YM was obtained for both AGEs, DOGDIC and glucosepane, with values increasing by between 3.0 and 8.5% (Fig. 2A and B). Upon cross-linking, all of the potential sites exhibit an increase in the YM which is independent of the AGE type, although the magnitude of the effect is very dependent on the local environment. The major contributor to the increase in YM is the increase in number of hydrogen-bonds present, typically three or more, between the AGE and the collagen peptide, which would otherwise be absent in the wild type collagen.

**Fig. 3:**
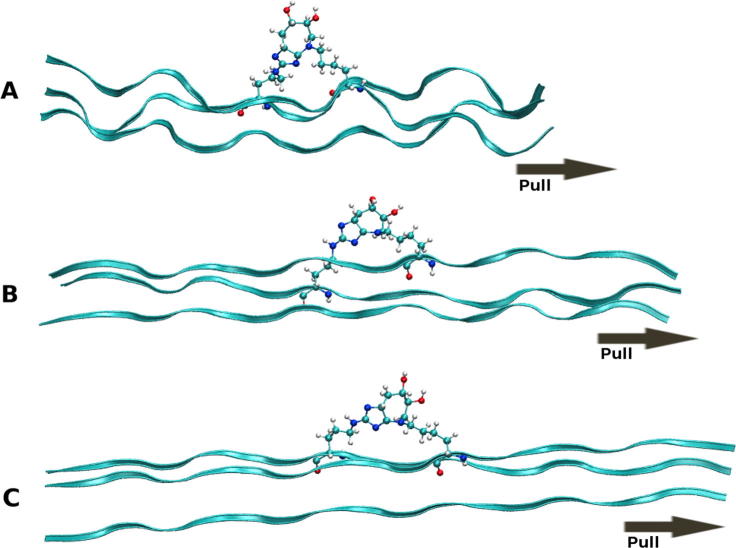
Series of three images showing the glucosepane cross-linked at G5; illustrating the starting structure (A), depicting the structure at 20% strain (B) and showing the final structure at 50% strain (C).

**Fig. 4:**
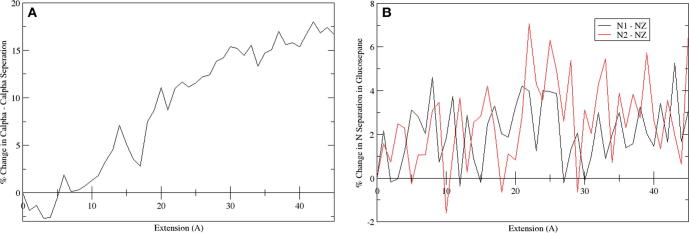
Plots showing the percentage increase in the separation between alpha carbon atoms in the backbone of the cross-linked lysine and arginine residues (A) and the nitrogen atoms within glucosepane (N1 and N2 from arginine and NZ from the lysine residue) (B).

At intermediate strains (20–40% strain), no statistically significant increase is observed in the tensile YM on introduction of an AGE between polypeptide chains (Fig. 2C and D). A decrease in YM, as seen for G4, G6, D1, D2 and D4, is likely the result of inaccuracies in the cross-sectional area of the peptide. In the intermediate strain domain, the hydrogen-bonds are no longer present due to the increased separation, and any change to the mechanics in this region would therefore be the result of covalently bonded interactions (Gautieri et al., 2009). No strain from the covalently bonded interactions occurred within this intermediate strain domain due to the load having been applied uniformly to all three chains. This results in an extension of the chains at an almost equal rate, as seen in Fig. 3, in such a way that the separation of the two cross-linked amino acids occurs at a gradual rate. Thus, the separation does not significantly increase beyond typical values until high strains near the fracture region. Fig. 4 shows this process graphically, with the straining of the cross-link not occurring until above a 25 Å extension (∼50% strain), as illustrated by the 15% difference in Cα-Cα separation. Even in this region of increasing Cα-Cα separation, the N1-NZ and N2-NZ separation does not increase dramatically. Based on the data presented in Fig. 4 and the final structure in Fig. 3, we suggest that the region where the YM would increase, as a result of direct straining of the covalent intra-molecular AGE cross-link bonds, would not be physiologically accessible, as the strain required would likely result in protein fracture. Thus, the observed tissue hardening should be the result of the influence of the cross-link within the low strain domain, which would have the most significant effect physiologically. Even in high strain tendons, which may experience strains of up to 16% (Stephens et al., 1989), the collagen molecule itself is thought to contribute only about 2% of the strain (Sasaki and Odajima, 1996b), the remainder resulting from stretching and sliding at the fibril, fibre, and fascicular level.

**Fig. 5:**
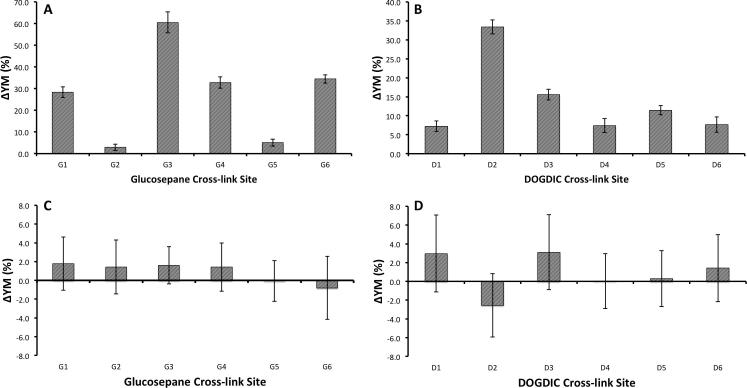
The percentage change in the lateral force-displacement ratio upon the formation of a Glucosepane (A, C) or DOGDIC (B, D) cross-link relative to the wild type collagen, in the low strain (A, B) region (0–15% strain) and in the intermediate strain (C, D) region (20–40% strain). The error bars illustrate the uncertainty as calculated by the standard error of the mean in the calculated values, for the 6 repeats. G (glucosepane) and D (DOGDIC) numbering corresponds to the numbering for sites given in Table 1.

We next calculated the lateral force-displacement ratio of the same collagen peptides to test whether this different loading mode is also affected by the presence of the AGE cross-links. A force-displacement ratio was used instead of the YM owing to difficulties in defining the cross-sectional area for lateral pulling. However, as we are using relative differences the results will be equivalent. The determination was carried out in both the low (Fig. 5A and B) and intermediate strain domains (Fig. 5C and D). Within the low strain domain we see a significant increase in the modulus with the introduction of an AGE cross-link. The relative increase in the modulus is much greater than was seen for the tensile modulus, with values increasing between 2.9 and 60.3%. As with the tensile modulus, the magnitude of the change is dependent on the local environment around the cross-link. Here the most significant contributor is the strong hydrogen-bonding occurring between the cross-link and the rest of the protein, which is removed upon application of the load to the polypeptide chain. This influence is seen to the greatest extent at sites G3 and D2 (Table 1), where the cross-link lies parallel to the principal axis of the collagen peptide, resulting in a 33% and 26.5% increase in the number of hydrogen-bonds within the peptide compared to the wild type collagen, which translates to a 60% and 33% increase in the moduli. Additionally, the presence of the cross-link lying parallel to the principal axis of the CLP, will likely result in an improvement in the Van der Waals packing, thereby further stabilising the initial structure to deformation.

**Table 1:** Locations of the previously identified intra-molecular glucosepane (G) (Collier et al., 2015) and DOGDIC (D) (Collier et al., 2016) crosslink formation sites. Column 1 gives site number, columns 2–4 gives the cross-linked residue pair (labelled using the UniProt residue number and triple helical residue number shown in brackets). Note that, with the exception of G5 and D5, G sites and D sites with the same number occur at different positions.

Cross-link	Chain α1 (a)	Chain α1 (b)	Chain α2
G1		^257^ARG(90)	^183^LYS(87)
G2	^527^LYS(360)		^456^ARG(360)
G3	^748^LYS(581)		^677^ARG(581)
G4	^958^LYS(791)	^956^ARG(789)	
G5	^1055^ARG(888)		^980^LYS(884)
G6	^1094^ARG(927)		^1020^LYS(924)
D1	^458^ARG(291)		^386^LYS(290)
D2	^734^ARG(567)	^731^LYS(564)	
D3	^958^LYS(791)		^884^ARG(788)
D4	^1025^ARG(858)	^1022^LYS(855)	
D5	^1055^ARG(888)		^980^LYS(884)
D6	^1085^LYS(918)	^1082^ARG(915)	

**Fig. 6:**
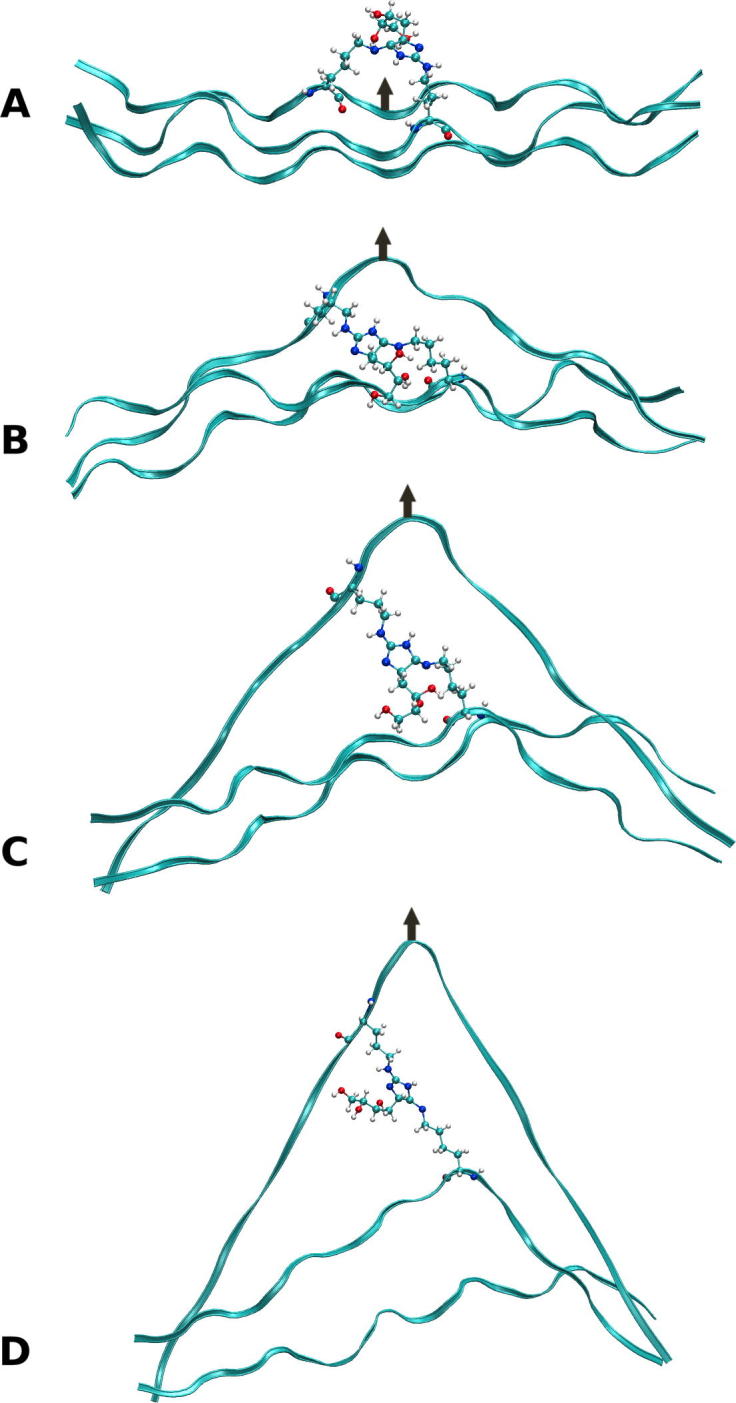
Series of four images showing the lateral pulling of DOGDIC cross-linked at site D5; illustrating the starting structure (A), depicting the structure at 10% (B), 35% strain (C) and showing the final structure at 50% strain (D).

As with the tensile modulus determined at intermediate strains, there is no statistically significant effect of cross-linking on the mechanical properties of the collagen peptides. One reason for this result is that all the hydrogen-bonds to the AGE have already been removed in the low strain domain and because the cross-links themselves are not strained heavily until the strain applied to the collagen molecule is above 50%, as seen in the final image (Fig. 6).

To date very few experimental studies have probed directly the role of AGE cross-linking on the mechanical properties of collagenous tissues. Reddy et al., conducted a macroscopic study probing the effect of pentosidine cross-links on the mechanical properties of a rabbit Achilles tendon, where they noted a significant 158% increase in the YM compared to a wild type sample (Reddy, 2004). Direct comparison between our study and that of Reddy et al. is not possible owing to the differences in the quantity and type of cross-link studied, as well as the scale of the collagen system studied. However, a qualitative comparison shows that in both studies the YM has increased due to the presence of AGE cross-links, thereby supporting the hypothesis that AGE cross-linking has a detrimental mechanical stiffening effect in collagenous tissues.

We have used steered molecular dynamics simulations to investigate the change in the mechanical properties of collagen in the presence of an AGE cross-link, specifically glucosepane and DOGDIC, testing both the tensile and lateral moduli at low (0–15% strain) and intermediate (20–40%) strains. After a comprehensive study of the 12 favourable cross-linking sites compared to the wild type, using six repeats, it was found that significant changes occurred to the mechanical properties in the low strain domain, whereas no significant changes within the uncertainty of the study were observed at intermediate strains. As in the energetics of AGE formation, the changes in mechanical properties, were influenced by the location within the collagen, specifically the capability of the cross-link to form hydrogen-bonding interactions with the neighbouring residues. The most significant changes in the YM occurred when the cross-link exhibited the largest number of hydrogen bonds to the peptide. Our results suggest that intra-molecular glucosepane or DOGDIC cross-linking may account for some of the stiffening of the collagen matrix however it is likely that a combination of intra- and inter-molecular cross-linking results in the large changes in YM observed.

## 3 Methodology

### 3.1 Collagen model

Sections of the triple helix collagen molecule were created, by selecting five or seven residues either side of the cross-linked residues, as listed in Table 1, to ensure minimum curvature or bending of the molecule at that point. Care had to be taken to ensure that the ends of the collagen sections created were as straight as possible, so that the pulling and fixed groups were close to linear and that the principal axis of the collagen was being measured for the tensile modulus, whilst avoiding any contributions to the mechanics from the bending stiffness (Bourne and Torzilli, 2011, Buehler, 2006). A number of different starting configurations were employed by producing the eleven collagen sections (or collagen-like peptides) at several different time points of the simulation of the entire collagen molecule, i.e. 37 ns, 41 ns, 45 ns, 49 ns, 53 ns and 57 ns, taken from our previous studies in which we identified the cross-linking sites (Collier et al., 2016, Collier et al., 2015). The heterogeneous nature of collagen means the absolute values will alter depending on the type of amino acid residues present, as hypothesised by Uzel and Buehler (Uzel and Buehler, 2009). It was therefore decided to provide relative (percentage difference) values calculated between the same sites in native collagen and the cross-linked collagen, thus removing the effects of the sequence on the mechanical properties.

Models were solvated fully using TIP3P water molecules with a 30 Å buffer from the solute (Jorgensen et al., 1983) and chloride counter ions were added where necessary to neutralise the system (Case et al., 2012). Appropriate charges were defined over the collagen to mimic collagen at a physiological pH. Each system underwent an energy minimisation of 2500 steps (500 steepest descent followed by 2000 conjugate gradient steps) followed by a 120 ps heating simulation from 0 K to 310 K at a heating rate of 2.5 K/ps. Finally, a 1 ns simulation was conducted within the NPT ensemble, restraining the complete protein for the first 500 ps, then restraining the backbone only for the following 250 ps, and finally the terminal backbone residues only during the final 250 ps. All restraints were implemented using a force constant of 75 kcal/(mol Å^2^).

### 3.2 Molecular dynamics

Molecular dynamics (MD) simulations of models encoded using the ff99SB forcefield were conducted using the Sander part of the Amber12 simulation package (Case et al., 2012). Water molecules were represented by the TIP3P model (Jorgensen et al., 1983). Coulombic potentials were calculated using the Particle Mesh Ewald summation with a cut-off radius of 8.0 Å (Darden et al., 1993). A time step of 2 fs was adopted for all MD simulations and hydrogen-bond lengths were constrained using the SHAKE algorithm (Ryckaert et al., 1977). Constant temperature and pressure were maintained with the Berendsen algorithm (Berendsen et al., 1984), using a barostat time constant of 5.0 ps atm^−1^ and a thermostat time constant of 1.0 ps. The number of hydrogen-bonds was calculated using the Hbonds plugin (version 1.2) within VMD, using an angle cut-off of 20° and distance of 3.0 Å (Humphrey et al., 1996).

### 3.3 Steered molecular dynamics

Owing to its steered molecular dynamics (SMD) functionality, excellent scalability and compatibility with the Amber force-field, NAMD version 2.11 (Phillips et al., 2005) was used for the SMD calculations. We used the same ff99SB force-field (Hornak et al., 2006) as in the MD simulations above, thus avoiding the need to re-parameterise the cross-links.

The tensile modulus was based on a similar SMD study by Lorenzo and Caffarena (2005), in which an external spring constant greater than 4 kcal/(mol Å^2^) and a pulling velocity of 0.1 Å/ps yielded optimum pulling conditions. The N-terminal nitrogen atoms of each of the three polypeptide strands were constrained. The centre of mass of the three carbon atoms at the C-terminus, defined as the SMD pulling group, was attached to a dummy atom via a virtual spring with a force constant of 7 kcal/(mol Å^2^). The dummy atom was then moved along the molecule’s principal axis at a constant velocity of 0.1 Å/ps. The resultant reactant force was recorded every 50 steps. The simulations were conducted for 80 ps and the resultant force displacement curve can then be used to obtain information on the stress-strain relationship and calculation of the YM (Lorenzo and Caffarena, 2005).

For the lateral investigation, the terminal carbon atoms at the C-terminus and nitrogen atoms of the N-terminus, of each of the three polypeptide chains were constrained at a position in space. The SMD pulling group is now defined as the alpha carbon atom of the central residue of an α1 cross-linked chain (in the case of the cross-link forming between the two α1 chains, the α1a chain was used). The centre of mass of the pulling atoms was attached to a dummy atom via a virtual spring with a force constant of 7 kcal/(mol Å^2^). The dummy atom was pulled at a constant velocity of 0.1 Å/ps and the resultant reactant force was recorded every 50 steps. The pulling vector in this case runs perpendicular to the principal axis of the collagen. The simulations were conducted for 50 ps.

The Young’s moduli for the systems were calculated in two strain regions, low strain (0–15%) and intermediate strain (20–40%). Additionally, as six repeats are conducted for each collagen section, with each beginning from different starting configurations, the mean average and the standard error of the mean were calculated and used in our comparison.
